# Postpartum reversible cerebral vasoconstriction with cortical subarachnoid hemorrhage and posterior reversible encephalopathy syndrome concomitant with vertebral artery dissection diagnosed by MRI MSDE method: A case report and review of literature

**DOI:** 10.1002/ccr3.6257

**Published:** 2022-09-12

**Authors:** Kenshi Sano, Atsushi Kuge, Rei Kondo, Tetsu Yamaki, Hiroshi Homma, Shinjiro Saito, Yukihiko Sonoda

**Affiliations:** ^1^ Department of Neurosurgery Yamagata City Hospital Saiseikan Yamagata Japan; ^2^ Department of Emergency Medicine Yamagata City Hospital Saiseikan Yamagata Japan; ^3^ Department of Neurosurgery Yamagata University, School of Medicine Yamagata Japan

**Keywords:** cervical artery dissection, reversible cerebral segmental vasoconstriction, postpartum, reversible posterior cerebral encephalopathy syndrome, motion‐sensitized balanced three‐dimensional turbo spin echo

## Abstract

Headache is one of the most common symptoms encountered during the postpartum period. The cause may be unknown, or the following illnesses are possible: cervical artery dissection (CAD), reversible posterior cerebral encephalopathy syndrome (PRES), and reversible cerebral vasoconstrictor syndrome (RCVS). It is suggested that they are interrelated and share a similar mechanism such as small vessel endothelial dysfunction, deficiencies in self‐regulation, and decreased sympathetic innervation of the posterior circulation. However, there are few reports of neuroradiological findings. We experienced a rare case of multiple postpartum vascular disease occurring at the same time. A 38‐year‐old woman suddenly developed thunderclap headache after giving birth. She was clear and had no neuropathy. Computed tomography revealed subarachnoid hemorrhage, including the cortical surface of the frontal lobe. Magnetic resonance image fluid‐attenuated inversion recovery revealed high‐intensity area in the bilateral basal ganglia and right occipital cortex. Angiography showed “string sausage” and extracranial left vertebral artery stenosis, but no aneurysm. Based on the clinical course and neuroradiological findings, we diagnosed her as postpartum vascular disease including CAD, PRES, RCVS, and cortical subarachnoid hemorrhage (SAH). Three‐dimensional black blood T1‐weighted images using a motion‐sensitized driven equilibrium three‐dimensional turbo spin echo (MSDE) sequencing method revealed an intramural hematoma consistent with the extracranial vertebral artery. After 3 months, MSDE lost its abnormal signal. Our case was rare in that multiple phenomena of postpartum vascular disease occurred at the same time. In particular, we could reveal that this speculation was reversible in the MRI MSDE sequencing.

## Graphical abstract

A case in which pain due to arterial dissection associated with postpartum period may have been involved in RCVS and PRES. And we confirmed its reversibility by MRI for understanding temporal changes in CAD, RCVS, and PRES, especially MSDE sequencing method.

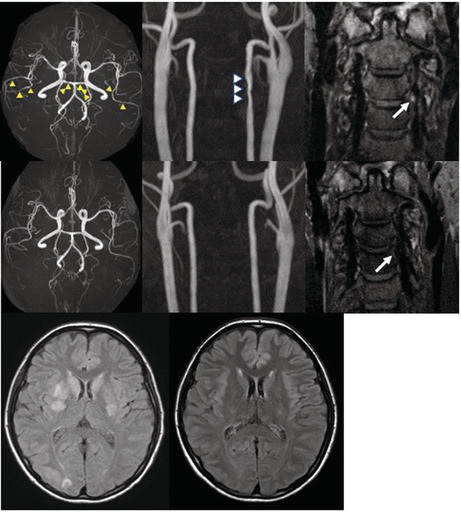

## 1 BACKGROUND

Headache is one of the most common symptoms that are encountered in the postpartum period.^1^ It may be attributable to dehydration, sleep deprivation, preeclampsia, among other etiologies. In contrast, more serious and life‐threatening conditions such as RCVS, SAH, and CAD have been reported as causes of postpartum headache.^2^, ^3^, ^4^ We experienced a rare case of multiple phenomena of postpartum stroke occurred at the same time and so we present a case report and a review of the literature.

## 2 CASE REPORT

**FIGURE 1:**
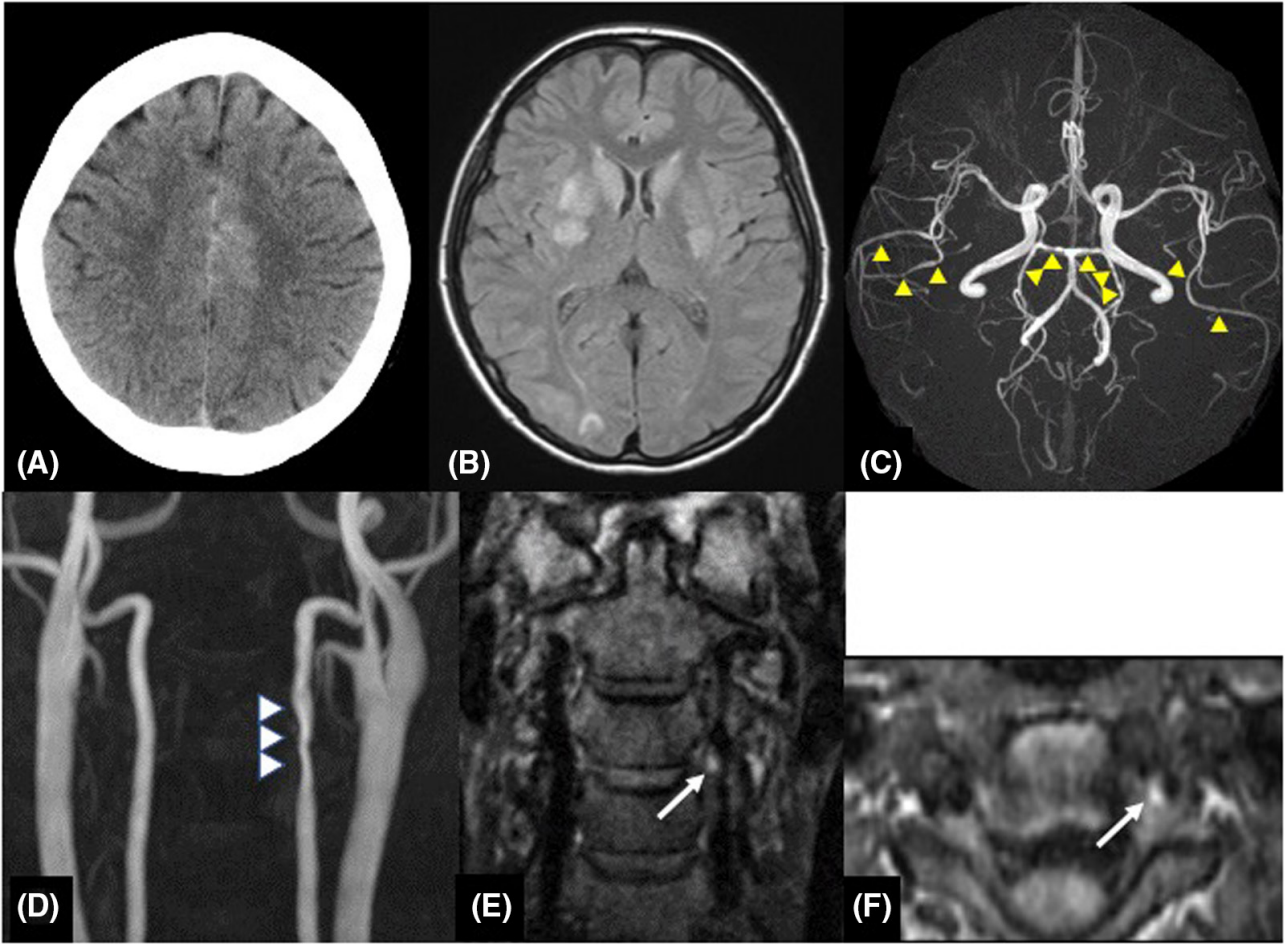
Neuroradiological findings at onset. (A) Plain CT scan revealed hyper density along the right cingulate sulcus, consistent with SAH. (B) MRI FLAIR images revealed hyperintensity involving the right occipital lobe and bilateral basal ganglia. These changes represent vasogenic edema due to PRES. (C) Magnetic resonance angiography (MRA) showed segmental narrowing (yellow arrowheads) of the bilateral middle and posterior cerebral arteries. (D) In right extracranial vertebral artery at the C3/4 level, cervical MRA (3D Time‐of‐flight) showed vessel contour abnormality (white arrowheads) and MSDE coronal (E) and axial (F) images showed a periluminal rim (white arrow) indicative of an intramural hematoma (IMH) suggesting a possible of dissection

**FIGURE 2:**
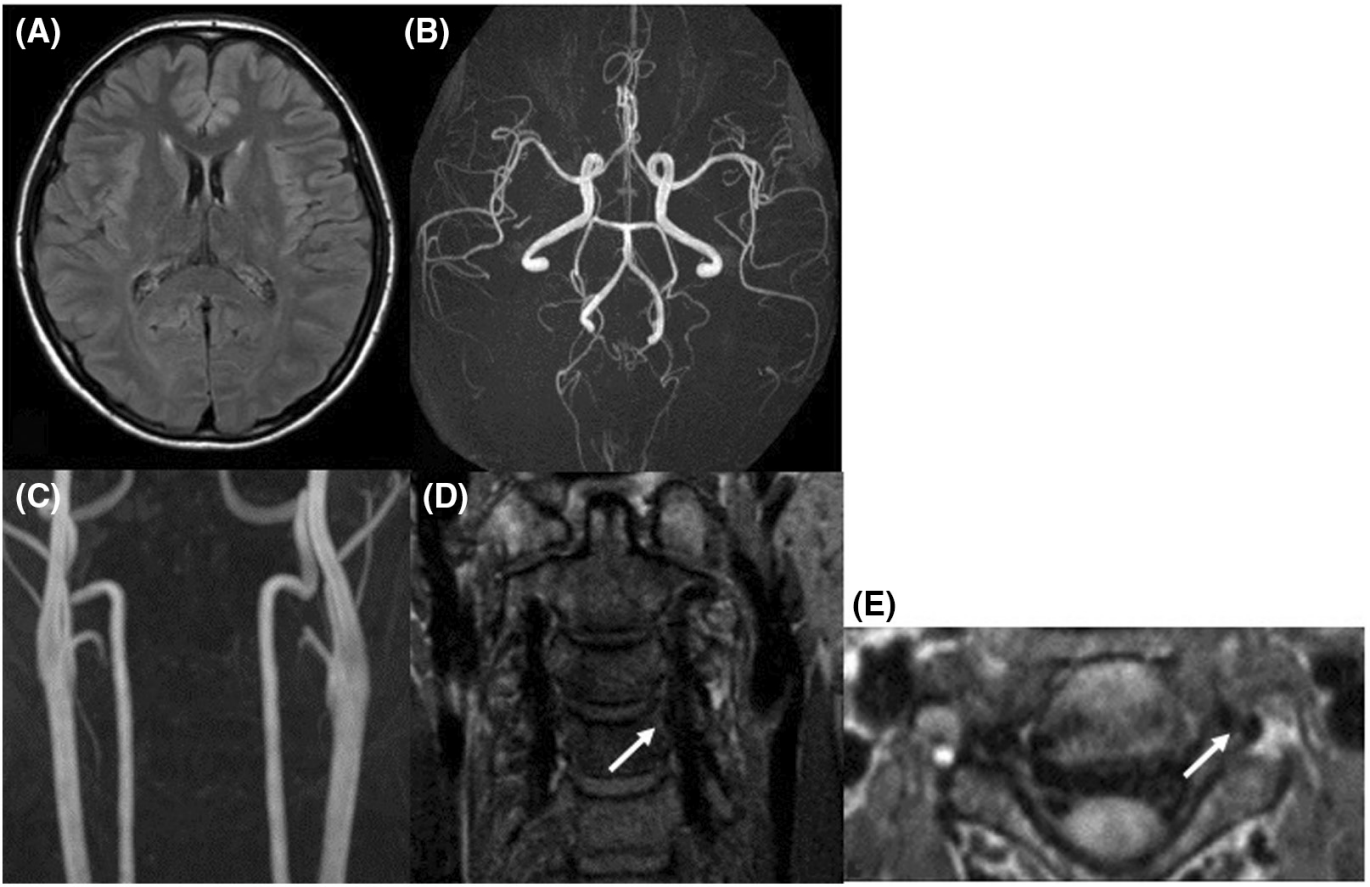
Neuroradiological findings 3 months after onset. (A) FLAIR images revealed the disappearance of initial abnormal lesions, and (B, C) MRA findings were normalized. MSDE coronal (D) and axial (E) showed the disappearance of a periluminal rim (white arrow). Based on the changes in findings over time, we diagnosed this lesion as vertebral artery dissection

A 38‐year‐old woman had a normal pregnancy and gave birth to her second baby by vaginal delivery without preeclampsia or other complications. She had no significant medical history, and there was no family history of aneurysmal SAH or migraine. She had no history of taking medicine. She initially had severe stiff shoulder and then begun to experience sudden thunderclap headaches with epigastric pain on the 18th day after delivery. At the time of visiting our emergency department, her consciousness was Glasgow Coma Scale E4V5M6 and had no cranial nerve impairment and motor‐sensory disturbance and other neurological deficits. The thunderclap headache and stiff shoulder improved. Laboratory investigations were all within normal limits, including blood cell count, liver enzyme levels, renal function and electrolytes and no coagulation abnormality. Urinalysis revealed no proteinuria. The neuroradiological findings at the onset were shown in Figure 1. Computed tomography (CT, Aquilion PRIME, Canon Medical Systems Corporation) showed interhemispheric subarachnoid hemorrhage covering the cortical surface of the frontoparietal lobe (Figure 1A). Magnetic resonance image (MRI, Achieva 3.0 T TX Quasar, Philips) fluid‐attenuated inversion recovery (FLAIR; repetition time (TR) = 13,000 ms, echo time (TE) = 140 ms, field of view (FOV) = 210 mm, matrix size = 512 × 512, slice thickness = 6 mm, slice gap = 0.6 mm, NSA = 1) revealed high‐intensity area in the bilateral basal ganglia and right occipital cortex (Figure 1B). Magnetic resonance angiography (MRA; TR = 25 ms, TE = 3.4 ms, FOV = 200 mm, matrix size = 512 × 512, slice thickness = 1.1 mm, gapless, NSA = 1, flip angle = 20‐degree, Scan technique = Inversion recovery) showed segmental narrowing and dilatation, called “sausage of the strings,” in the bilateral middle and posterior cerebral artery (Figure 1C). Cervical MRA showed irregular stenosis of the left vertebral artery (VA) at the level of third‐fourth cervical vertebra (Figure 1D). Neither infarction nor brain aneurysm was detected. In the evaluation of the three‐dimensional black blood T1‐weighted imaging using the motion‐sensitized driven equilibrium three‐dimensional turbo spin echo (MSDE; TR = shortest, TE = shortest, FOV = 230 mm, matrix size = 512 × 512, slice thickness = 0.7 mm, gapless, NSA = 2, flip angle = 90‐degree, Scan technique = spin echo) sequence method, coronal (Figure 1E) and axial view (Figure 1F) revealed a periluminal rim, called crescent sign, suggested an intramural hematoma (IMH) consistent with the extracranial vertebral artery stenosis area (Figure 1E,F). We speculated that RCVS, PRES, and CAD occurred almost at about the same time. We treated her with rest and intensive hypotension without antithrombotic therapy. After discharge, her clinical course was good with no progression. After 3 months, CT and MRI revealed the disappearance of SAH and FLAIR high‐intensity legion (Figure 2A). MRA showed improvement of segmental narrowing (Figure 2B). Cervical MRA revealed normal left VA (Figure 2C) and MSDE (Figure 2D,E) showed no abnormal signal. These results indicate that these conditions were reversible, and we have judged these reversible lesions as multiple phenomena of postpartum stroke occurred at the same time.

## 3 DISCUSSION

RCVS, which has been described in the literature since the early 1970s, is characterized by severe headache (especially called thunderclap headache), with or without other acute neurological symptoms, and diffuse segmental vasoconstriction of cerebral arteries, which is reversible within 3 months.^5^ It is common in postpartum women and called postpartum cerebral angiopathy.^3^, ^6^ Previous studies revealed that 5%–12% of RCVS patients are postpartum women.^7^, ^8^ As neuroradiological findings of RCVS, in one third to a half of cases reveal cortical SAH, reversible cerebral edema called PRES, cervicocarotid dissection (CAD), intraparenchymal hemorrhage, subdural hemorrhage, and ischemic stroke.^3^, ^9^, ^10^, ^11^, ^12^ Cortical SAH is the most frequent finding of RCVS and was reported to account for 30%–38% of hemorrhagic complications.^8^, ^9^ Cortical SAH associated with postpartum angiopathy could result from rupture or reperfusion injury affecting the small arteries of the leptomeninges.^13^ The diagnosis of RCVS should be considered in patients who present with recurrent thunderclap headache, with or without focal neurological signs and non‐aneurysmal SAH.^14^ PRES is one of the hypertensive encephalopathies that can cause cerebral edema, seizures, and both ischemic and hemorrhagic strokes.^15^ It was reported that approximately 4% of 95 women with postpartum headache were diagnosed with PRES.^1^ Bartynski et al. reported that the parietal and occipital lobes were the most frequently abnormal regions in 134 patients of PRES, but lesions of basal ganglia, such as our case, were found in 14% of the series.^16^

It is suggested that RCVS and PRES are interrelated and share pathophysiologic mechanisms such as small vessel endothelial dysfunction, deficiencies in self‐regulation, and decreased sympathetic innervation of the posterior circulation.^3^, ^10^, ^14^, ^15^ In one prospective study of 77 consecutive male and female patients with RCVS, 7 (9.1%) developed PRES.^10^

CAD is a major cause of cerebral ischemia and hemorrhage in young adult,^17^ but it is a rare complication during the postpartum period. The presence of intramural hematoma (IMH) is the basis of the diagnosis of CAD.^18^ IMH, which can be identified on MSDE, characteristically has a crescentic shape adjacent to the vessel lumen and often spirals along the length of the artery.^18^, ^19^, ^20^ Previous reports suggested that the MSDE‐3D‐TSE sequence was useful for assessing vessel walls in the dissection and aneurysms.^21^, ^22^, ^23^ MSDE causes phase dispersion of blood spin using a magnetic field to suppress blood flow signal, producing 3D T1‐weighted images.^24^ Digital subtraction angiography (DSA) is the gold standard for diagnosis and follow‐up of CAD; however, it is invasive and cannot determine IMH.^17^ Other angiographic features are luminal flap, false lumen, long tapered stenosis, and dissecting aneurysms.^17^ We were able to confirm the presence of intramural hematoma and the process of its change, so we have diagnosed with CAD on MRI‐MSDE method.

To the best of our knowledge, 26 women and 35 dissections have described postpartum patients with CAD, including our patient (Table 1).^25^, ^26^, ^27^, ^28^, ^29^, ^30^, ^31^, ^32^, ^33^, ^34^, ^35^, ^36^ The mean age was 35 years (range, 26–44 years); the mean time from birth to onset was 10.5 days (range, 1 h–53 days). For CAD‐affected lesions, 15 (57%) women had a single lesion and the other women (43%) had multiple lesions. Among the 26 women with CAD, 6 (23%) had SAH, 6 (23%) had RCVS, and 9 (34%) had infarction. One case had PRES based on FLAIR high‐intense area in the bilateral occipital cortex.^34^ Previous reports indicate that 8–12% of RCVS patients have CAD.^13^, ^37^ Wiebers et al. described the first case of a 44‐year‐old woman with postpartum CAD in 1985.^25^ She developed infarction in the left MCA territory and was treated with heparin. The first CAD‐related RCVS case was diagnosed incidentally.^38^ Arnold et al. reported that 6% of 102 postpartum women with CAD.^30^ Interestingly, the prognosis was good with or without antithrombotic therapy.

**TABLE 1:** Characteristics of postpartum women with CAD

	Age	Postpartum(days)	Peripartum blood pressures	Mode of delivery	SAH	RCVS	Ischeimc	Dissection					Antithrombotic therapy	mRS
								Lt IC	Rt IC	Lt VA	Rt VA			
Wiebers & Mokri (1985)^18^	44	6	normal	vaginal	No	Yes	Yes	Yes	No	No	No	Single	Yes	1
Sharshar et al. (1995)^19^	NA	NA	NA	NA	No	No	No	No	No	No	No	NA	NA	NA
Brunix et al. (1996)^20^	33	14	NA	NA	No	No	Yes	No	No	No	No	NA	NA	NA
Gasecki et al. (1999)^21^	34	6	Normal	Vaginal	No	No	Yes	Yes	No	No	No	Single	Yes	6
Gasecki et al. (1999)^21^	34	14	Normal	Vaginal	No	No	No	No	No	No	Yes	Single	Yes	0
Gasecki et al. (1999)^21^	36	14	Normal	Cesarean	No	No	Yes	Yes	No	No	No	Single	Yes	1
Gasecki et al. (1999)^21^	26	9	Normal	Vaginal	No	No	Yes	No	Yes	No	No	Single	Yes	NA
Abisaab et al., 2004^22^	35	9	NA	Cesarean	No	No	No	Yes	Yes	No	No	Multiple	NA	NA
Arnold et al. (2008)^23^	38	7	NA	NA	yes	yes	no	no	no	yes	yes	multiple	NA	NA
Arnold et al. (2008)^23^	41	18	NA	NA	No	No	No	No	No	Yes	No	Single	NA	NA
Arnold et al. (2008)^23^	35	5	NA	NA	No	Yes	Yes	Yes	No	No	Yes	Multiple	NA	NA
Arnold et al. (2008)^23^	27	11	NA	NA	No	Yes	No	No	No	No	Yes	Single	NA	NA
Arnold et al. (2008)^23^	34	7	NA	NA	No	No	No	No	No	No	Yes	Single	NA	NA
Arnold et al. (2008)^23^	38	8	NA	NA	Yes	No	No	No	Yes	No	No	Single	NA	NA
Hesieh et al. (2008)^24^	32	12	Elevated	Cesarean	Yes	No	No	No	Yes	No	Yes	Multiple	No	0
Baffour et al. (2012)^25^	34	14	Elevated	Vaginal	No	No	Yes	Yes	Yes	No	No	Multiple	Yes	0
Drazin et al. (2012)^13^	37	0	NA	NA	No	No	No	No	No	Yes	Yes	Multiple	No	NA
Kelly et al. (2014)^26^	39	11	Normal	Vaginal	No	No	No	Yes	Yes	Yes	No	Multiple	Yes	0
Kelly et al. (2014)^26^	39	24	Elevated	Vaginal	No	No	No	No	No	Yes	Yes	Multiple	Yes	0
Kelly et al. (2014)^26^	29	53	Elevated	Vaginal	No	No	No	Yes	No	Yes	Yes	Multiple	Yes	0
Kelly et al. (2014)^26^	28	4	Elevated	Cesarean	Yes	No	No	Yes	No	No	No	Single	Yes	0
Kelly et al. (2014)^26^	32	0	Normal	Vaginal	No	No	Yes	No	Yes	No	No	Single	Yes	1
Nishimura et al. (2015)^27^	35	11	Normal	Na	No	Yes	No	No	No	No	Yes	Single	No	0
Garrard et al. (2015)^28^	35	10	NA	NA	Yes	No	No	No	No	No	Yes	Single	Yes	NA
Finley et al. (2015)^29^	36	21	Elevated	NA	No	No	Yes	No	No	Yes	No	Single	NA	NA
Present case	38	18	Normal	Vaginal	Yes	Yes	No	No	No	Yes	No	Single	No	0

Abbreviations: IC, internal carotid artery; Lt, left; mRS, modified Rankin Scale; NA, not available; RCVS, reversible cerebral vasoconstriction syndrome; Rt, right; SAH, subarachnoid hemorrhage; VA, vertebral artery.

According to our case and previous reports, we speculate that postpartum vascular lesions are a series of pathological conditions. Although not confirmed in our case, RCVS and CAD are associated with alteration of the vessel wall of the vasa vasorum, vasculopathy‐related arterial tears, and genetic predisposition.^6^, ^10^, ^39^, ^40^ RCVS and PRES share a vulnerability in the posterior circulation.^10^, ^39^ These pathologies have been speculated to include small vessel endothelial dysfunction, defective autoregulation, and reduced sympathetic innervation of the posterior circulation.^10^, ^39^, ^41^ In some pathological reports, skin biopsy of CAD patients revealed ultrastructural derangements of type I and type III collagen fibrils.^42^, ^43^ Another study including superficial arterial biopsy of CAD patients showed degradation with inflammatory and erythrocytic accumulation between the tunica media and adventitia.^44^

In pregnancy and postpartum periods, endothelial damage associated with sudden changes in blood pressure led to the destruction of the blood–brain barrier.^5^, ^45^ In addition, increased activity of the immune system can activate proteolytic enzymes such as matrix metalloproteinases and cause weakening of the arterial wall.^44^ A series of conditions may indicate arterial wall instability and dysfunction. Our case began with stiff shoulders and then became aware of thunderclap headache. From this mode of onset, CAD first occurred, and we speculated that RCVS and PRES could be induced by being aware of stiff shoulders and pain.

## 4 CONCLUSION

Our case was rare in that multiple phenomena of postpartum stroke occurred at the same time. What is remarkable about this case was the confirmation of the reversibility of these pathological conditions through multifaceted evaluations, including the MSDE sequencing method. In addition, these pathologies were the same properties, are reversible, and show a good prognosis with hypotension and rest treatment, without antithrombotic therapy.

## AUTHOR CONTRIBUTIONS

Atsushi Kuge: Conceptualization, Writing–review and editing. Rei Kondo: Conceptualization, Supervision. Tetsu Yamaki: Visualization. Kazuki NAKAMURA: Data curation. Shinjiro Saito: Supervision, Writing–review and editing. Yukihiko Sonoda: Supervision.

## CONFLICT OF INTEREST

None declared.

## ETHICAL APPROVAL

This study with the use of human tissue was in accordance with the ethical standards of the declaration of Helsinki with its latest revision in 2004. Informed consent: obtained.

## CONSENT

Written informed consent was obtained from the patient to publish this report in accordance with this journal's patient consent policy.

## Data Availability

The data that support the findings of this study are available on request from the corresponding author. The data are not publicly available due to privacy or ethical restrictions.
